# Attention Called to a Most Invaluable Medicine

**Published:** 1901-02

**Authors:** N. F. Howard

**Affiliations:** Dahlonega, Ga.


### ATTENTION CALLED TO A MOST INVALUABLE MEDICINE.

BY N. F. HOWARD, M.D.,

DAHLONEGA, GA.

In the autumn of 1883, I jumped some five feet above the ground from the top of a falling box, and caught upon my feet, and received a heavy shock. At once there was a piercing pain that passed through my bladder, apparently near its neck. In a few moments I was compelled to void the contents, and had frequently to do so. The next day I rode nine miles to see a patient, and did not discover till the morning thereafter that I was suffering with hemorrhage from my bladder. I then made my way home as best I could, and though I suffered pain from the time of receiving the injury of the organ and had frequent calls to void the contents thereof, yet until that morning I did not think that there was any hemorrhage but only a strain of the parts that

I was suffering from. I found that my trip had complicated my case, and so I determined to be quiet in my room, and take such medicine as I thought would hasten my recovery, such as aromatic sulphuric acid, astringents, anodynes, etc., as was thought best by myself and other physicians of our town. Yet for some days I found no relief. I could feel, as it seemed to me, the hemorrhage flowing into my bladder, as from a laceration; then I would have to void it off, and then it would often appear as pure blood unmixed with the urine. I would have to obey this call every few hours. As I did not improve for a day or two I became very much interested in the case, as well as in the patient, and began to investigate to find out what Dr. Watson said in his "Lectures" would be the most effective remedy for internal hemorrhage; and fortunately found on page 903 the very medicine I sought after, which is extract of gum rhatany. This medicine taken every four hours in scruple doses proved a panacea to me in my affliction, from the very first dose, both in the arrest of the hemorrhage and the relief from pain; so that in twelve hours the urine gave a purple tinge, as if you had mashed up a few poke berries in it. I continued the rhatany in smaller doses occasionnally for a few days till there was an entire disappearance of any sanguinary coloring in the contents voided. After a week I began to walk around the streets a little. In a week or two after I rode a short distance in the buggy; whenever I struck a rough place in the road I was compelled to get out and lead my horse over such places, on account of the pain the jolting of the buggy gave me. This convinced me more than ever that there must have been laceration near the neck of the bladder. So I gave up buggy riding for some weeks to come. I then felt, as I do to-day, that this lost-sight-of and much neglected and most valuable medicine had not been appreciated by the profession as it deserved to be. I had up to that time prescribed gum catechu and kino as the vegetable astringents in cases needing such medicines, and had given no attention to the rhatany, as I found a bottle with a half pound of the gum in it, not opened. For a dozen years, from the year 1883, I used this medicine in quite a number of cases with those who were troubled with internal hemorrhages, both active and passive, with satisfactory results; yet

I have no record of the cases treated. The last case I remember to have treated, some five years ago, was a lady friend who was suffering with passive hemorrhage of the kidneys, and she was entirely relieved in two or three days by means of extract of rhatany. Up to within a few weeks ago I thought the profession generally were using this valuable medicine, but I found that I was mistaken. A friend of mine came to me for medicine for passive hemorrhage of the kidneys. I discovered that I had no rhatany, as my supply was exhausted, not having been in active practice for three or four years. We then sought it at the drug store here and at Gainesville and at Atlanta, and could not obtain it. Then Dr. George of Gainesville, sent to Park, Davis & Co. of Detroit, to get the medicine for me. In that way, therefore, I discovered that the profession was not using this valuable medicine.

Brethren, I have written this much to call attention to the intrinsic value of this medicine; and if you will revive the use of it you certainly will not be disappointed in its results in cases of internal hemorrhage to which the human body is subject.

